# Family-oriented and family-centered care in pediatrics

**DOI:** 10.1186/1824-7288-35-12

**Published:** 2009-05-12

**Authors:** Massimo Pettoello-Mantovani, Angelo Campanozzi, Luigi Maiuri, Ida Giardino

**Affiliations:** 1 University of Foggia School of Medicine, Institute of Pediatrics, Foggia, Italy; 2 World Health Policy Forum (WHPF), Giessen, Germany; 3 University of Foggia School of Medicine, Center of Laboratory Medicine, Foggia, Italy

## Abstract

**Background:**

To humanize the management of children in hospitals has become a serious concern of civil society and one of the main goals of public and private health centers, health care providers and governments.

**Discussion:**

The concepts of family-centered and family-oriented care are discussed with the aim to emphasize their importance in pediatrics. Notions related to family-centered care, such as cultural diversity and cultural competence, are also discussed given the importance they have gained following the recent transformations of socioeconomic, demographic and ethnic characteristics of economically advantaged Countries. Family-centered care has developed as a result of the increased awareness of the importance of meeting the psychosocial and developmental needs of children and of the role of families in promoting the health and well-being of their children. Family-oriented care aims at extending the responsibilities of the pediatrician to include screening, assessment, and referral of parents for physical, emotional, social problems or health risk behaviors that can adversely affect the health and emotional or social well-being of their child.

**Summary:**

Family-centered and family-oriented care concepts should be incorporated into all aspects of pediatricians' professional practice, whether it is private practice or in public hospitals, to better serve the needs of ill children.

## Background

During the last fifty years there has been a profound change in the approach to children who are hospitalized whether for short or long periods of time [1-9]. In fact, to humanize the management of children in hospitals has become a serious concern of civil society and one of the main goals of public and private health centers, health care providers and governments [10].

The concept of family-centered care has developed initially in the economically advantaged countries. It was the result of an increased social awareness, which focused particularly on the importance of meeting the psychosocial and developmental needs of children, with an emphasis on the role of families in promoting the health and well-being of their children [1-9]. Family-centered care in pediatrics (Appendix 1) is based on the understanding that the family is the child's primary source of strength and support and that the child's and family's perspectives and information are important in clinical decision making [11]. It is now a consolidated notion for family-centered trained professionals, that health care experiences can enhance parents' confidence in their roles, and eventually increase the competence of children and young adults to take responsibility for their own health care. Mostly in anticipation of the transition to adult service systems [12,13]. In particular, family-centered care has been promoted during the last fifteen years as the philosophies, principles and practices that put the family at the heart or center of services; the family has been identified as the driving force [14].

In parallel, the notion of *family pediatrics *(family-oriented care) developed among pediatricians as a concept not in contrast but in harmony although different from family-centered care. As recently emphasized by the report of the American Academy of Pediatrics (AAP) Task Force on the Family, *family pediatrics *aims at extending the responsibilities of the pediatrician to include screening, assessment, and referral of parents for physical, emotional, social problems or health risk behaviors that can adversely affect the health and emotional or social well-being of their child [11].

This paper, will debate the concepts of family-centered care and family pediatrics with the aim to emphasize the importance of the beneficial interfacing between the two conceptions. Furthermore, the unprecedented, rapid transformations of socioeconomic, demographic and ethnic characteristics of western societies and their cultural repositioning in the global context, has brought to public attention other important aspects and notions related to family-centered care and family pediatrics. Including cultural diversity and cultural competence, which will also be discussed in the paper.

Public policies established by federal, state, and local governing and administrative bodies clearly have a great impact on the health, safety, and welfare of children and their families and on the ability of pediatricians to serve them. The organization of pediatric care show profound differences and divergent problems throughout the world, particularly between economically advantaged and challenged Counties. However, significant differences exist also among the economically developed Countries. For instance, in the US although the concepts of family-oriented and family-centered care emerged and developed to become a consolidated part of its health systems since over fifty years, the AAP observes that the US stands apart from most similarly developed countries in having no coherent set of public policies that would create a social context supportive of families, traditional or otherwise [11]. In Europe, a recent study of the Union of National European Paediatric Societies and Associations (UNEPSA) emphasized that the diversity of pediatric care in different countries in Europe is extreme and the differences are difficult to be analyzed and harmonized [15]. In a further study [16], UNEPSA also investigated the status of primary pediatric care in Europe. Data from the UNEPSA study were obtained form 34/48 European (EU and non-EU) countries, including Turkey, Switzerland, and Israel, which covered over 85% of the European population by serving approximately 158 million children who were younger than 15 years. Twelve of the 34 countries had a system for primary care for children, 6 had a general practitioner/family doctor system, and 16 had a combined system for the care of children who are younger 15. Three countries (Bulgaria, Croatia, and Czech Republic) were reported to have a public health reform in progress, including a shift from the pediatric system to the combined system. Three countries (8%) did not have any pediatricians working at the primary level of care. Fourteen countries (41%) reported having community pediatricians.

A most interesting case seems to be that regarding Italy, where family-pediatrics and family-pediatricians may be considered respectively equivalent to the Italian Public Health Institution "*Pediatria di Famiglia*" and to the professional figure of *"Pediatra di Famiglia*" also called "*Pediatra di Libera Scelta*", which in Italy is part of the public health system. Furthermore, most recently the Italian Ministry of Health, the major health care provider in that Country, made an effort to explore the possibility to establish the pediatric "*Casa della Salute*", which was designed to be an institution somehow comparable to the Medical Home typical of the Anglo-Saxon medical system.

The paper aims at emphasizing the importance of positively interfacing family-centered care and family-oriented care concepts in pediatrics, also suggesting that most favorable environments to practice an effective family-centered care in pediatrics should be provided by health care institutions.

## Discussion

### From the concept of Medical Home to Family-centered care

Historically, family-centered care has evolved from the concept of Medical Home, which is not a building, house, or hospital, but rather an approach to providing comprehensive primary care. A Medical home is defined as a primary care that is accessible, continuous, comprehensive, family centered, coordinated, compassionate, and culturally effective. In a medical home, a pediatric clinician works in partnership with the family/patient to assure that all of the medical and non-medical needs of the patient are met [13]. Through this partnership, the pediatric clinician can help the family/patient access and coordinate specialty care, educational services, out-of-home care, family support, and other public and private community services that are important to the overall health of the child/youth and family. As a matter of fact, for many years family-centered care had the characteristic of an effective medical home [12] and much of the early work focused on hospitals. For instance, as a multitude of studies progressively emphasized the impact of separating hospitalized children from their families, many institutions adopted policies that welcomed family members to be with their child around the clock and also encouraged their presence during medical procedures.

In Europe, such new concepts and policies gradually became accepted, although only few research studies and extensive reports, frequently of humanistic rather than scientific type, were produced on the topic. Conversely, in the Anglo-Saxon Countries family-center care has long been a matter of public attention and scientific investigation. In the New World, family-centered care was given further bust by consumer-led movements of the 1960s and 1970s and by professionals in education, health, and child development, which prompted US Federal legislation of the late 1980s and 1990s, much of it targeted at children with special needs, to provide additional validation of the importance of family-centered principles [13].

### Family-centered care concepts merging in family pediatrics

Nowadays, attention for family-centered care continues to grow in economically advantaged Countries and its momentum is further supported by a growing body of research and by prestigious organizations. For instance, the American Academy of Pediatrics (AAP) has incorporated some of the principles of family-centered care into its policy statements [14] and the US Institute of Medicine in its 2001 report *Crossing the Quality Chasm: A New Health System for the 21st Century*, emphasized the need to ensure the involvement of patients in their own health care decisions, to better inform patients of treatment options, and to improve patients' and families' access to information [17].

The embedding of family-centered care concepts in family-oriented care in pediatrics has produced a positive new approach to health care which shapes health care policies, programs, facility design, and day-to-day interactions among patients, families, physicians, and other health care professionals [18]. However, the practice of family-centered care by health care professionals could be productive only if the community of health care professionals recognized the vital role that families play in ensuring the health and well-being of children and family members of all ages. In that view, it is critical that health practitioners acknowledge that emotional, social, and developmental support are integral components of health care. Furthermore, each child and family's innate strengths should be respected and the health care experience must be considered as an opportunity to build on these strengths and support families in their care-giving and decision-making roles [19].

### Core principles of family-centered care

Under many aspects, family-centered care can be considered as an extension of patient-focused care, a concept that acquired general attention in the early 1990s [8]. The fundamental premise of patient-focused care was to abandon the traditional approach in which care was based on what worked well from an organizational perspective, promoting the concept that delivery of care should be based on the needs of the patient. Family-centered care simply takes patient-focused care to the next step and expands the concern to include in the loop of care those persons who are important in a patient's life [13].

It is now a consolidated convincement in pediatrics that acceptance and correct practice of family-centered approaches will produce better health outcomes and wiser allocation of resources as well as greater patient and family satisfaction. However, much confusion remains over what family-centered critical care actually is [8]. Many clinicians incorrectly associate family-centered care with open visiting. This misconception originates, in part, from the common implementation of policies for flexible visiting hours in units that are attempting to provide more family-oriented care. Given that family-centered care is a philosophical approach to care that recognizes the needs of patients' family members as well as the important role that family members play during a patient's illness, it is therefore not defined by a singular intervention. In fact, no single intervention and not even a group of interventions will ensure a family-focused environment. For instance, it would be wrong to believe that simply allowing a family member to be at a patient's bedside 24 hours a day would mean that the staff met the family's needs. In fact, having a family member present in a situation in which staff members are not equipped to meet the family's needs could ultimately have unfavorable consequences. Family members may be more stressed if they feel ignored by a nurse, neglected by physicians or are made to believe that they are somehow in the way or interfering with the patient's care [8].

An important step toward the establishing of family-centered care concepts has been made in 2003 by the American Academy of Pediatrics, which issued a policy statement indicating nine key principles, that should guide the practice of family-centered care in pediatrics (Appendix 2). The AAP statement indicates that family-centered care is grounded in collaboration among patients, families, physicians, nurses, and other professionals for the planning, delivery, and evaluation of health care as well as in the education of health care professionals. This partnership proposed by the AAP statement implies that practice of family-centered care in pediatrics should pursue a number of unavoidable tasks, including the support of youth as they transition to adulthood, acknowledgment of family as the constant in a child's life and the honor of cultural diversity and family traditions. Such tasks are reported in Appendix 3.

The 2003 AAP statement has been undoubtedly a significant step toward the correct management of the needs of hospitalized children. However, in spite of the widespread attention and growing knowledge regarding family-centered care, there is still confusion over this concept and its practice may cause frustrations for many staff members who think that family-centered care may not be in the best interest of either patients or health personnel. In general, adult patients who are able should always be asked to what extent they want their family to participate in care. Family's involvement is presented as a choice and lets patients know that family members are welcome should the patients so decide [8]. Patients may, in fact, not want any visitors or any information given out to family members. Of course, in pediatrics the practice of these concepts is facilitated by the nature of the patients and their dependence by families or guardians. However, it is also important that care for the needs of children and family's involvement are not misrepresented as opportunities for the staff to alleviate their job or to charge families with duties or responsibilities that do not pertain to them.

On the other hand it is equally important and helpful for staff members to see that the essence of family-centered care is consistent with patient-centered care. To such regard, a great concern often raised by staff members is that family-centered care demands that staff relinquish all structures within the unit that allow some form of order in this otherwise chaotic environment. This concern should be considered absolutely not the case. For example, during a critical illness, patients' families will benefit from guidance and structure to help them deal with the situation. It is crucial that staff members fully understand what family-centered care is and is not, to avoid any room for disruptive discussions, as well as they must be reassured by knowing that boundaries and limitations are still in place and that the expertise of staff members remains a critical factor in ensuring the success of family-centered care [8,13].

The important concept that must be reiteratively stressed is that the needs of the children are always the priority, even in a family-centered environment. Research indicates that it is important to the hospitalized child's family members to be assured that he/she is receiving the best possible care [20]. Interventions such as having family members present during procedures and resuscitations help to reassure family members that everything possible is being done for the patient [21]. Understanding and meeting children's needs in hospitals should always be the priority for both the patient's family and health care providers.

Finally, family-centered way of practice requires that outdated rules and regulations that were imposed for the benefit of the organization rather than children or their families should be reviewed and reconsidered. Structures and policies that provide for the support and safety of patients and their family members are generally welcomed by family members and help staff members to carry out their responsibilities in a timely and efficient manner [8].

### Importance of Cultural Diversity and Cultural Competence for family-centered care in the context of socioeconomic, demographic and ethnic changes

Cultural Diversity is generally defined by coexistence of numerous distinct ethnic, racial, religious, or cultural groups within one social unit, organization, or population. As a source of exchange, innovation and creativity, cultural diversity is as necessary for humankind as biodiversity is for nature. In this sense, it is the common heritage of humanity and should be recognized and affirmed for the benefit of present and future generations. This approach to cultural diversity should be taken by economically advantaged Countries in confronting socioeconomic, demographic and ethnical changes within their civil societies. In our increasingly diverse societies, Cultural Diversity is essential to ensure harmonious interaction among people and groups with plural, varied and dynamic cultural identities as well as their willingness to live together [22-24].

Cultural competence is defined as a set of values, behaviors, attitudes, and practices within a system, organization, program or among individuals which enables them to work effectively cross culturally (Appendix 4). Further, it refers to the capability to respect the beliefs, language, inter-personal styles and behaviors of individuals and families receiving services, as well as staff who are providing such services. At a systems, organizational, or program level, cultural competence requires a comprehensive and coordinated plan that includes interventions at all the levels from policy-making to the individual, and is a dynamic, ongoing, process that requires a long-term commitment. An important component of cultural competence is linguistic competence, the capacity of an organization and its personnel to communicate effectively, and convey information in a manner that is easily understood by diverse audiences including persons of limited local language proficiency, those who are not literate or have low literacy skills, and individuals with disabilities.

Cultural Competence and Cultural Diversity are profoundly interconnected to the concept and practice of family-centered care. In fact, Cultural Competence is necessary in providing care to culturally diverse families. Family-centered care values the strengths, cultures, traditions and expertise that everyone brings to a respectful family/professional partnership, where families feel they can be decision makers with providers at different levels, in the care of their own children and as advocates for systems and policies supportive of children and youth with special health care needs. It requires culturally competent attitudes and practices in order to develop and cultivate those partnerships and to have the knowledge and skills that will enable the health care practitioners to be "family-centered" with the many diverse families that exist and they interact with. Various and untraditional strategies can also be adopted to support health system in providing proper care to sick children, including those who are hospitalized. For instance, building relationships with community cultural brokers, is an approach that can help health care institutions and health care professionals in understanding rules and behaviors of different communities [25-29].

In brief, knowledge of cultural diversity is vital at all levels of health system and knowledge about cultures and their impact on interaction with health care is essential for health care professionals, whether they are practicing in a clinical setting, education, research or administration. Knowledge and skills related to the important concept of cultural diversity and acquaintance with cultural competence are factors that must be considered strategic to strengthen and broaden health care delivery systems.

## Summary

In conclusion, the social, political and most in general cultural changes in today's globalized world, has accelerated various innovative approaches in different sectors of society, including the health system. Although Family-centered care applies to patients of all ages, and it may be practiced in any health care setting, this unique approach is of particular importance in the management of hospitalized children, given the role that hospitalization may play as a time of potential crisis for the child and family [11].

Family-centered care should be integrated in the management of children in teaching and non teaching hospitals; and education and training in family-centered care should be provided to all trainees, students, and residents as well as staff members [30].

In accordance with the American Academy of Pediatrics'recommendations [13], core concepts of family-centered care should be incorporated into all aspects of pediatricians' professional practice, whether it is private practice or in hospitals (Appendix 5). Whatever the context, pediatricians should unequivocally convey respect for parents' or guardians' unique insight into and understanding of their child's behavior and needs, should actively seek out their observations, and should appropriately incorporate family preferences into the care plan. Decisions on a patient's plan of care should be made only after such consultation has been made.

Finally, the most favorable environments to practice an effective family-centered care in pediatrics must be provided by health care institutions. They should design their facilities to promote the philosophy of family-centered care and in hiring of staff, developing job descriptions, and designing performance-appraisal processes, they should make explicit the expectation of collaboration with patients and families and other family-centered behaviors.

## Competing interests

DECLARATION: All the authors declare that they have no competing interests as they have been described in the "manuscript sections for Debate Articles" of the IJP.

## Authors' contributions

All the Authors equally contributed to the article. In particular, they have equally made substantial contributions to conception and design, or acquisition of data, or analysis and interpretation of data; furthermore, they have been involved in drafting the manuscript or revising it critically for important intellectual content; Finally, all authors read and approved the final manuscript.

## Appendix 1

### Family-Centered Care: Definition and its Importance in Pediatrics

• ***Family-centered care***. An innovative approach to the planning, delivery, and evaluation of health care that is grounded in mutually beneficial partnerships among health care patients, families, and providers. It incorporates the patient, the health care provider, and the family in all aspects of care.

• ***Heart of Pediatric family-centered care***. The belief that health care providers and the family are partners, working together to best meet the needs of the child. Parents and family members provide the child's primary strength and support. Their information and insights can enhance the professional staff's technical knowledge, improve care and help design better programs and friendlier systems

^13 ^*Neff JM et al. Pediatrics – 2003*

## Appendix 2

### Core Principles of Family-Centered Care

1. Respecting each child and his or her family

2. Honoring racial, ethnic, cultural, and socioeconomic diversity and its effect on the family's experience and perception of care

3. Recognizing and building on the strengths of each child and family, even in difficult and challenging situations

4. Supporting and facilitating choice for the child and family about approaches to care and support

5. Ensuring flexibility in organizational policies, procedures, and provider practices so services can be tailored to the needs, beliefs, and cultural values of each child and family

6. Sharing honest and unbiased information with families on an ongoing basis and in ways they find useful and affirming

7. Providing and/or ensuring formal and informal support (eg, family-to-family support) for the child and parent(s) and/or guardian(s) during pregnancy, childbirth, infancy, childhood, adolescence, and young adulthood

8. Collaborating with families at all levels of health care, in the care of the individual child and in professional education, policy making, and program development

9. Empowering each child and family to discover their own strengths, build confidence, and make choices and decisions about their health

^11 ^*American Academy of Pediatrics – 2003*

## Appendix 3

### Tasks of Family-Centered Care in Pediatrics

#### Family-centered care

1. Acknowledges the family as the constant in a child's life.

2. Builds on family strengths.

3. Supports the child in learning about and participating in his/her care and decision-making.

4. Honors cultural diversity and family traditions.

5. Recognizes the importance of community-based services.

6. Promotes an individual and developmental approach.

7. Encourages family-to-family and peer support.

8. Supports youth as they transition to adulthood.

9. Develops policies, practices, and systems that are family-friendly and family-centered in all settings.

10. Celebrates successes.

Sources

• ^31 ^National Center for Family-Centered Care: *Family-Centered Care for Children with Special Health Care Needs*. Bethesda, MD: Association for the Care of Children's Health; 1989

• ^32 ^Bishop et al.: *Family/Professional Collaboration for Children with Special Health Care Needs and their Families*. Burlington, VT: University of Vermont, Department of Social Work; 1993.

• ^33 ^Bishop et al: *Family-Centered Care Projects 1 and 2 (2002–2004)*. Algodones, NM: Algodones Associates; 2004

## Appendix 4

### Principles of Cultural Competence

#### An organization should

1) Value diversity in families, staff, providers and communities;

2) Have the capacity for cultural self-assessment;

3) Be conscious of the dynamics inherent when cultures interact, e.g. families and providers;

4) Institutionalize cultural knowledge; and

5) Develop adaptations to service delivery and partnership building reflecting an understanding of cultural diversity.

#### An individual should

1) Examine one's own attitude and values;

2) Acquire the values, knowledge, and skills for working in cross cultural situations; and

3) Remember that every one has a culture.

Sources

• ^34 ^Maternal and Child Health Bureau (MCHB): *Guidance and Performance Measures for Discretionary Grants, Health Resources and Services Administration*. Washington, DC: U.S. Department of Health and Human Services; 2004.

• ^23 ^Cross T, Bazron B, Dennis K, Isaacs M: *Towards a culturally competent system of care*. Washington, DC: Georgetown University Child Development Center, CASSP Technical Assistance Center; 1989

• ^35 ^Goode J: *Definition of Linguistic Competence*. Washington, DC: National Center for Cultural Competence; 2004.

## Appendix 5

### Strategies for an Effective Family-Centered Care in Hospital and Private Practice

• Conduct attending physician rounds (ie, patient presentations and rounds discussions) in the patients' rooms with the family present should be standard practice. This will facilitate the exchange of information between the family and other members of the child's health care team and encourage the involvement of the family in the decisions that are commonly made during rounds. In teaching hospitals, a lasting impression will be made on students and house staff when they are encouraged in this process by their attending physician.

• Invite parents and guardians to be present with their child during medical procedures and offered support before, during, and after the procedure. Working with families in decision making and information sharing in all practice settings should always take into account the older child's and young adult's capacity for independent decision making and right to privacy and confidentiality.

• Promote the active participation of all children in the management and direction of their own health care, beginning at an early age and continuing into adult health care. During their work in collaboration with families and other health care professionals, pediatricians should also examine systems of care, individual interactions with patients and families, and patient flow and should modify these as needed to improve the patient's and family's experience of care.

• Share information with children and families in ways that are useful and affirming in every health care encounter

• Encourage and facilitate family-to-family support and networking, particularly with families of similar cultural and linguistic backgrounds or families who have children with the same type of medical condition.

• Invite the families to collaborate in pediatric research programs. Families should have a voice at all levels in shaping the research agenda, in determining how children and families participate in research, and in deciding how research findings will be shared with children and families

• Create opportunities for children and families to serve as advisors in family advisory councils, committees, and task forces dealing with operational issues in hospitals, clinics, and office-based practices; as participants in quality improvement initiatives; as educators of staff and professionals in training; and as leaders or co-leaders of peer support programs.

^11 ^*American Academy of Pediatrics – 2003*

^13 ^*Neff JM et al. Pediatrics – 2003*
